# The integrase of genomic island GI*sul2* mediates the mobilization of GI*sul2* and IS*CR*-related element CR2-*sul2* unit through site-specific recombination

**DOI:** 10.3389/fmicb.2022.905865

**Published:** 2022-08-01

**Authors:** Gang Zhang, Qinna Cui, Jianjuan Li, Ruiliang Guo, Sébastien Olivier Leclercq, Lifeng Du, Na Tang, Yuqin Song, Chao Wang, Fangqing Zhao, Jie Feng

**Affiliations:** ^1^State Key Laboratory of Microbial Resources, Institute of Microbiology, Chinese Academy of Sciences, Beijing, China; ^2^College of Life Science, University of Chinese Academy of Sciences, Beijing, China; ^3^School of Life Sciences, Zhengzhou University, Zhengzhou, China; ^4^UMR ISP, INRAE, Université François Rabelais de Tours, Nouzilly, France; ^5^Beijing Institutes of Life Science, Chinese Academy of Sciences, Beijing, China

**Keywords:** integrase, genomic island (GI), mobilization, ISCR elements, site-specific recombination

## Abstract

In the worldwide health threat posed by antibiotic-resistant bacterial pathogens, mobile genetic elements (MGEs) play a critical role in favoring the dissemination of resistance genes. Among them, the genomic island GI*sul2* and the IS*CR*-related element CR2*-sul2* unit are believed to participate in this dissemination. However, the mobility of the two elements has not yet been demonstrated. Here, we found that the GI*sul2* and CR2*-sul2* units can excise from the host chromosomal attachment site (*attB*) in *Shigella flexneri*. Through establishing a two-plasmid mobilization system composed of a donor plasmid bearing the GI*sul2* and a trap plasmid harboring the *attB* in *recA*-deficient *Escherichia coli*, we reveal that the integrase of GI*sul2* can perform the excision and integration of GI*sul2* and CR2*-sul2* unit by site-specific recombination between *att* core sites. Furthermore, we demonstrate that the integrase and the *att* sites are required for mobility through knockout experiments. Our findings provide the first experimental characterization of the mobility of GI*sul2* and CR2*-sul2* units mediated by integrase. They also suggest a potential and unappreciated role of the GI*sul2* integrase family in the dissemination of CR2*-sul2* units carrying various resistance determinants in between.

## Introduction

**Figure 1:**
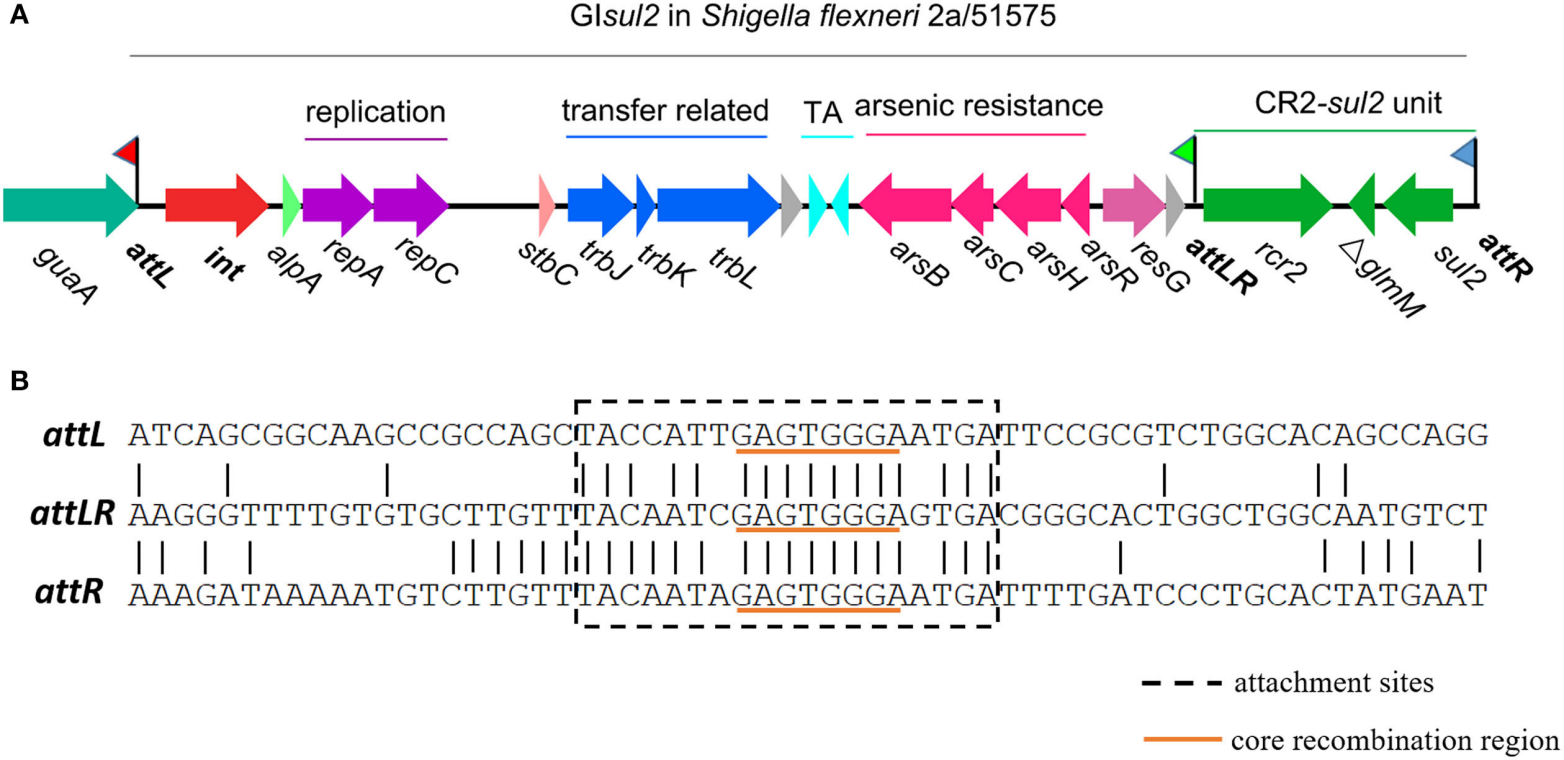
Genetic organization of genomic island GI*sul2* in *S. flexneri* 2a str. 2457T (accession AE014073) or *S. flexneri* 51575 (accession AZPD01000036, position 2108-17567). **(A)** Organization of the GI*sul2* element. The thick central line represents the backbone of the GI*sul2* with flags representing the three attachment sites (*attL/attLR/attR*). Horizontal arrows indicate the position, size, and orientation of ORFs with the names below. The *guaA* gene encoding a guanosine monophosphate synthetase (GMP) is shown in teal. The *int* gene encoding the integrase is represented in red. Bright green, light red, and pink ORFs denote *alpA, stbC*, and *resG* genes, respectively. Genes putatively involved in replication, transfer-related, toxin-antitoxin system, arsenic resistance, and the CR2*-sul2* unit are shown in purple, blue, cyan, pink and green, respectively. Gray ORFs encode hypothetical proteins. **(B)** Nucleotide alignment of *att* sites of GI*sul2*. The dotted box represents the expected *att* sites recognized by the integrase. The orange underlined motif indicates the putative core recombination region.

Mobile genetic elements (MGEs), including genomic islands (GIs), play a major role in the evolution of bacteria, and more specifically in the wide dissemination of antibiotic resistance genes (ARGs) (Juhas et al., 2009; Hall, 2010; Carraro and Burrus, 2014; Partridge et al., 2018; He et al., 2019; Halder et al., 2022; Li et al., 2022). Among them, GI*sul2* was proposed to participate in the dissemination of sulphonamide resistance gene *sul2* (Nigro and Hall, 2011; Harmer et al., 2017). GI*sul2* was first detected in the chromosome of *Enterobacter cloacae* subspecies cloacae type strain ATCC 13047, in the chromosome of *Shigella flexneri* ATCC 700930 (also known as 2457T), and in the large conjugative plasmid pAB3 of *Acinetobacter baumannii* ATCC 17978 (Nigro and Hall, 2011). GI*sul2* was then also reported in the chromosome of *A. baumannii* ATCC 19606 and plasmids of several γ-proteobacteria (Hamidian and Hall, 2016; Harmer et al., 2017). GI*sul2* is usually inserted into the 3′ end of chromosomally-encoded *guaA* genes (GMP synthase) at the 5′-GAGTGGGA-3′ integration site (Harmer et al., 2017). GI*sul2* and its derivatives are also frequently found as part of antibiotic resistance island B (ARI-B) of IncC plasmids (Anantham et al., 2015; Harmer and Hall, 2015), but in this case, the integration site is limited to 5′-GGGA-3′, which may correspond to a secondary integration site (Harmer et al., 2017). According to the recent genomic description (Harmer et al., 2017), the element consists of genes putatively involved in mobilization (excision/integration, replication, and transfer), followed by a toxin–antitoxin system, an arsenic resistance operon, a CR2 element, and the *sul2* resistance gene (Figure 1).

The first common region (CR) element, CR1, was discovered in the 3′-conserved segment (3′-CS) of some class 1 integrons, where it is associated with antibiotic resistance genes not embedded in gene cassettes and is usually found between partial duplications of the 3′-CS (Stokes et al., 1993; Partridge and Hall, 2003). Other CR elements were later identified in numerous bacteria, especially in Gram-negative pathogens, and are frequently linked to diverse ARGs (Partridge and Hall, 2003; Toleman et al., 2006a; Schleinitz et al., 2010; Fang et al., 2019). CR2 is found downstream of the *sul2* gene in a head-to-head organization with a truncated *glmM* gene in between, a structure found at the 3′ end of GI*sul2*, and hereafter referred to as the CR2-*sul2* unit (Figure 1). More commonly, CR2-*sul2* units carry various other ARGs in between and locate on other MGEs. Recently, CR2 was also reported to likely contribute to the mobilization of the high-level tigecycline resistance gene *tet*(X) in *Escherichia coli* (Fang et al., 2019; He et al., 2019; Sun et al., 2019). CR elements are proposed to move by a process called rolling-circle replication because of the distant similarity of their encoded gene to the transposases of the IS*91* family insertion sequences, known to promote their own spread through this mechanism (Toleman et al., 2006b). Accordingly, the term IS*CR* was proposed for these elements (Toleman et al., 2006a). CR element boundaries, called *ter*IS and *ori*IS for 5′ and 3′ boundaries, respectively, were inferred from their genetic sequence for most elements, but not all (Toleman et al., 2006a; Lallement, 2018). The lack of experimental demonstration for a *rcr* (coding region of CR)-mediated rolling circle mechanism and the yet unclear boundaries of some CR elements make the hypothesis of CRs being IS elements still debatable. We therefore conservatively keep the CR denomination in this study to define these genetic elements.

Despite the numerous occurrences of GI*sul2* and CR2-*sul2* units in various bacteria, suggesting horizontal gene transfer, their mobility has not yet been demonstrated. GI*sul2* mobilization was tested in an *E. coli* genetic context, but no excised circular intermediate could be detected (Harmer et al., 2017). In the present study, we demonstrate that GI*sul2*-encoded integrase mediates (i) the excision of GI*sul2* and CR2-*sul2* from their original chromosomal location, (ii) the integration of GI*sul2* and CR2-*sul2* in two-plasmid mobilization assay system in *E. coli recA*-deficient mutant. All these reactions are site-specific and rely on the presence of GI*sul2* attachment (*att*) sites.

## Results

### GI*sul2* and CR2*-sul2* unit can excise from the chromosome of *S. flexneri*

**Figure 2:**
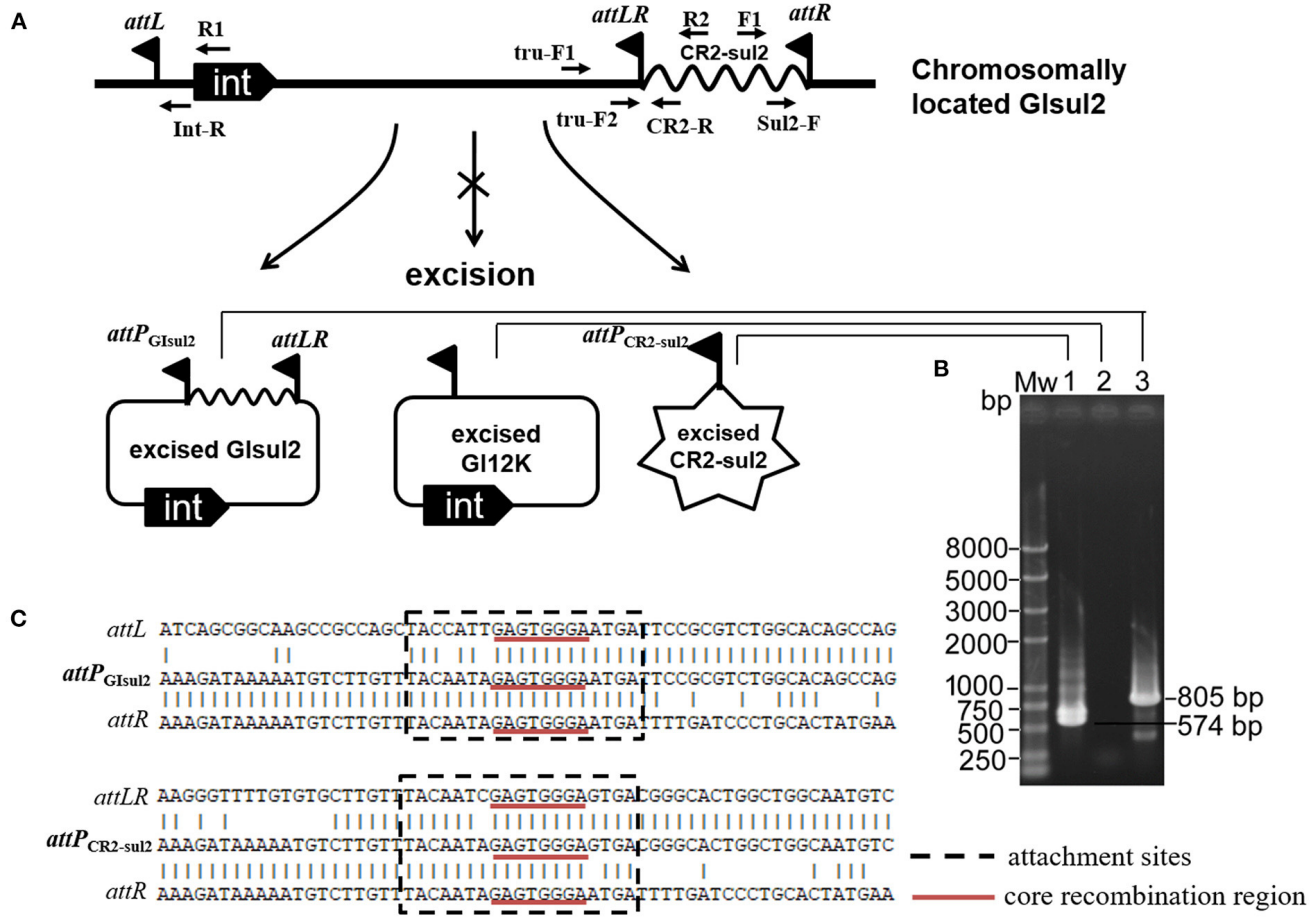
Excision of GI*sul2* and of CR2*-sul2* unit from the chromosome of *S. flexneri* 51575. **(A)** The predicted circular intermediates resulting from recombination between the three *att* sites of GI*sul2*. The crossed arrow indicates that the event could not be observed. Solid flags denote the various *att* sites. **(B)** Nested PCR assays for the detection of the three potential circular intermediates: Mw, M5 DL2000 plus DNA Marker (Mei5 Biotechnology, Beijing); lane 1, CR2*-sul2* circular intermediate; lane 2, GI12K circular intermediate; lane 3, GI*sul2* circular intermediate. **(C)** Alignment of the sequenced junctions resulting from the circularization of GI*sul2* (*attP*_GIsul2_) and of CR2*-sul2* unit (*attP*_CR2−sul2_) with their corresponding initial *att* sites.

By analyzing in detail the structure of GI*sul2* reported in the reference sequence of *S. flexneri* 2a 2457T (GenBank accession AE014073), we found that three putative *att* sites were present in this island. In addition, to the already described *attL* and *attR* located at the left and right end of the GI (Harmer et al., 2017), a third *att* site, termed *attLR*, lies 112–130 bp upstream of the *rcr2* gene (Figure 1A). The three sites are 19 bp-long and they all share the common central nucleotide motif 5′-GAGTGGGA-3′ (Figure 1B), previously proposed to be their core recombination site (Harmer et al., 2017). Interestingly, the putative *ter*IS proposed for CR2 (5′-GGGAGTGACGGGCACTGGC-3′), which is the putative 5′ boundary of the element (Toleman et al., 2006a), starts in the middle of the core recombination region of *attLR* (Figure 1B). According to the possible recombination events between the putative *att* sites, Figure 2A shows the three predicted circular intermediates that could be produced, namely, excised GI*sul2* (site-specific recombination between *attL* and *attR*), excised CR2-*sul2* (site-specific recombination between *attLR* and *attR*), and an excised 12-kb segment named GI12K (site-specific recombination between *attL* and *attLR*). We first tested whether any of these circular intermediates could be naturally excised from the chromosome of *S. flexneri* 51,575. This strain carries a single copy of GI*sul2* almost identical to that of *S. flexneri* 2a 2457T (GenBank accession number AZPD01000036, position 2,108–17,567), except for a 140-bp region in the *trbL* gene with several differences. GI*sul2* in *S. flexneri* 51,575 is also inserted in the 3′ end of *guaA* gene, resulting in the same three *att* sites as in Figure 1.

The presence of the junction region of each predicted circular intermediate was tested through PCR amplification on crude DNA extracts from overnight culture in standard conditions using a nested procedure because of the very low amplification yield after the first round of PCR. Two pairs of nested primers F1/R1 and Sul2-F/Int-R were used for the detection of the GI*sul2* circular form (805 bp), two pairs of nested primers F1/R2 and Sul2-F/CR2-R were used for the detection of the CR2*-sul2* unit circular form (574 bp), and two pairs of nested primers tru-F1/R1 and tru-F2/Int-R were used for the detection of the GI12K circular form (767 bp; Figure 2A, Supplementary Table 1). These various PCR reactions resulted in only two target-size products (Figure 2B). The first corresponded to the junction in the circular form of GI*sul2*, resulting from the recombination between *attL* and *attR* sites. The second corresponded to the junction in the circular form of CR2-*sul2* unit, resulting from the recombination between *attLR* and *attR* sites. Sequencing of the amplified junctions confirmed that they consisted of *attL/attR* and *attLR/attR* hybrids, hereafter named *attP*_GIsul2_ and *attP*_CR2−sul2_, respectively (Figure 2C, Supplementary Figure 1), indicating that GI*sul2* and CR2*-sul2* units are naturally excised from the chromosome of *S. flexneri* 51575.

### A donor plasmid bearing GI*sul2* can integrate into the chromosomal *attB* site downstream of *guaA* gene in *recA*-deficient *E. coli*

**Figure 3:**
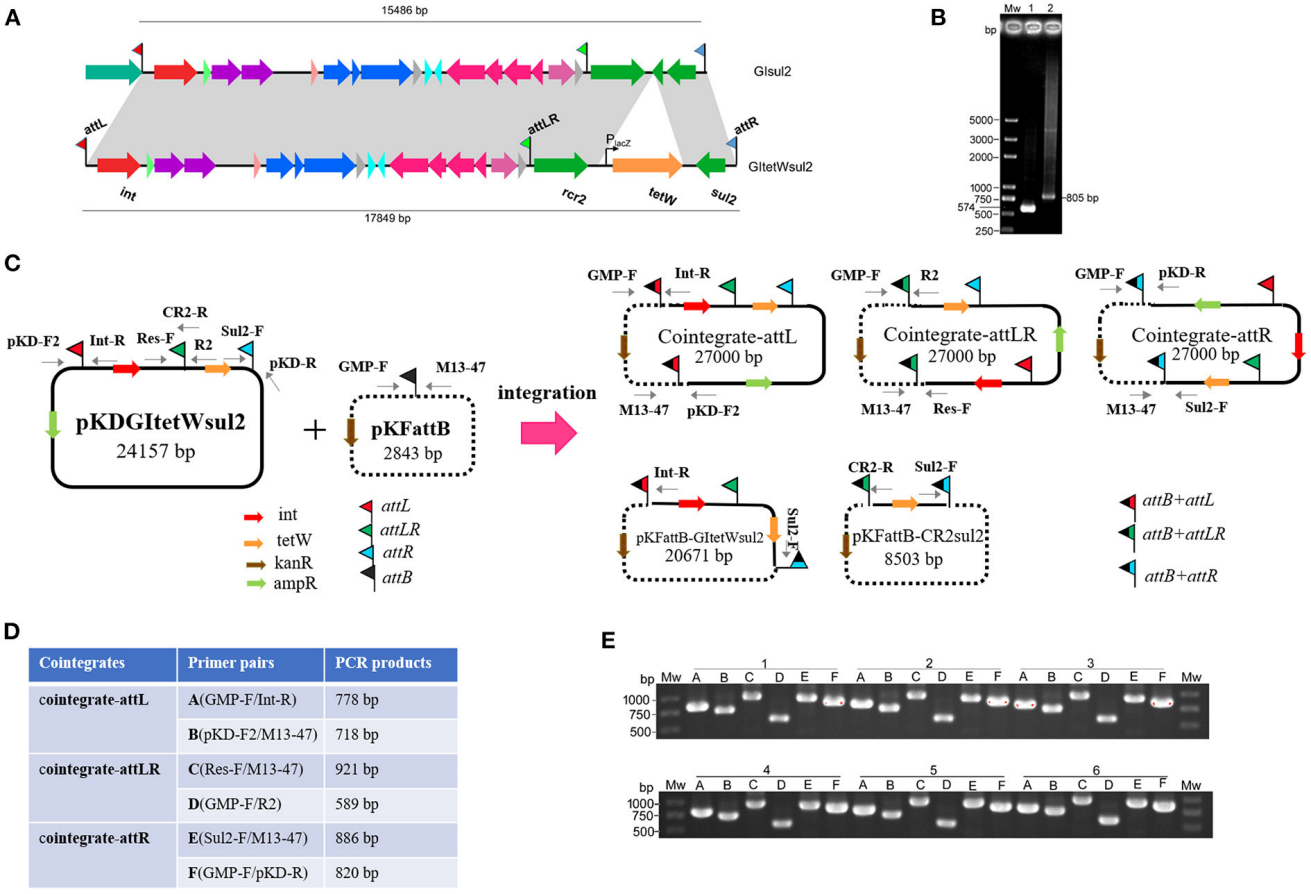
Co-integrate formation in *E. coli* DH5α through site-specific recombination at the *att* sites. **(A)** Construction of GI*tetWsul2*. The gray shadings denote 100% identity between GI*tetWsul2* and the original GI*sul2*. ORFs (horizontal arrows) are colored as in Figure 1. **(B)** Detection of circular intermediates excised from the donor plasmid pKDGItetWsul2 in *E. coli* DH5α. Mw, DNA size marker; lane 1, CR2*-sul2* unit circular intermediate; lane 2, GI*tetWsul2* circular intermediate. **(C)** Organization of the donor (pKDGItetWsul2) and trap (pFKattB) plasmids used in the two-plasmid integration experiment, and expected recombinant structures. Intervening ORFs are depicted with filled arrows while red, green, blue, and black flags denote *attL, attLR, attR*, and *attB*, respectively. The putative hybrid attachment sites resulting from site-specific recombination are represented by two-color flags. Primers used for the detection of the various structures are indicated. **(D)** Couples of primer pairs used to detect co-integrate structures in recombinant bacteria, and their expected amplification size. **(E)** PCR amplification using the six primer pairs from **(D)** on the plasmid extracts of six independent recombinant colonies. Colonies are labeled 1–6, with the six primer pairs amplification **(A–E)** displayed below each of them.

To avoid excision mediated by RecA-dependent homologous recombination and the difficulty of genetic operation in *Shigella*, pKDGItetWsul2 (24,157 bp) carrying a modified version of GI*sul2*, as a donor plasmid, was established and introduced into the *recA*-deficient strain, *E. coli* DH5α. pKDGItetWsul2 was constructed from pKD46, a temperature-sensitive plasmid that can be eliminated at 37 or 42°C (Datsenko and Wanner, 2000). pKDGItetWsul2 carries an ampicillin resistance marker and GI*sul2* in which Δ*glmM* between *rcr2* and *sul2* genes was replaced by a constitutively expressed *tetW* gene (tetracycline resistance; Figure 3A, Supplementary Table 2). PCR assays targeting *attP*_GIsul2_ and *attP*_CR2−sul2_ were then performed on *E. coli* DH5α (pKDGItetWsul2) as in Section Introduction, and the amplified products (validated by sequencing) confirmed that the excision of GI*tetWsul2* and CR2-*sul2* from donor plasmid can still occur in a *recA*^−^ genetic context (Figure 3B).

Since *E. coli* DH5α carries a GI*sul2*-empty *attB* site at the 3′ end of its *guaA* gene, we tested the integration potential of the excised elements into this chromosomal position. *E. coli* DH5α (pKDGItetWsul2) culture was grown overnight at 42°C to discard pKDGItetWsul2 and then transferred at 42°C on fresh LB plates containing tetracycline to select for colonies bearing chromosomally integrated circular intermediates. Twelve randomly selected resistant colonies were tested using the same six pairs of primers as in Figure 3D, except that the primer GMP-R replaced the vector-specific primer M13-47 (Supplementary Figure 2). Only Sul2-F/GMP-R and GMP-F/pKD-R PCR reaction provided an amplification (Supplementary Figure 2). Successful amplification was obtained in all colonies for the primer pairs, corresponding to the recombination between *attR* of pKDGItetWsul2 and *attB*. Sequencing of the PCR products confirmed the expected junction. The observations indicate that the chromosomal *attB* site can recombine with a plasmidic *attR* site in a *recA*^−^ genetic context, leading to donor plasmid carrying GI*sul2* integration into the chromosome.

### The donor plasmid, GI*sul2*, and CR2-*sul2* unit can integrate into the *attB* site of a trap plasmid in *recA*-deficient *E. coli*

To clearly demonstrate the mobilization mechanism for GI*sul2* and CR2-*sul2* units, we applied a two-plasmid assay system consisting of the donor plasmid and trap plasmid. A similar strategy was also used for studying integration mechanisms of pathogenicity islands in *E. coli* 536 (Wilde et al., 2008). The trap plasmid, pKFattB (2843 bp), was constructed by cloning an uninterrupted *attB* region (811 bp) into the high copy vector pKF18k-2 (Supplementary Table 2). This plasmid bears a kanamycin resistance gene (kanR) as the selective marker. The two plasmids pKDGItetWsul2 and pKFattB were co-transformed into *E. coli* DH5α. The correct clones were performed using the two-plasmid mobilization assay. Based on the GI*sul2* and CR2-*sul2* excisions observed above, we speculated that there could be five types of integrations: integration of the GI*tetWsul2* circular intermediate into the trap plasmid (pKFattB*-*GItetWsul2 in Figure 3C), integration of the CR2-*sul2* intermediate (pKFattB-CR2sul2 in Figure 3C), and three possible integrations of the whole donor plasmid, one for each *att* site (cointegrate-attL/-attLR/-attR, Figure 3C).

First, we tested for the integration of the whole donor plasmid. There are three *att* sites in the donor plasmid, and we, therefore, designed six specific primer pairs (A to F) targeting each of the possible recombined sites (junctions; Figure 3D). Gel bands corresponding to the PCR product of every junction were detected in all 12 randomly selected recombinant plasmid extracts used as templates (six shown, Figure 3E). Sequencing of the PCR products confirmed that the amplification indeed corresponded to the recombination between *attB* and each of the three *att* sites (*attL, attLR*, and *attR*). To understand which type of integration dominated in each colony, we used *Bam*HI to digest the recombinant plasmids. Two bands of 15.7 and 11.2 kb, respectively, corresponding to the digestion of the donor plasmid integration through *attR*-*attB* recombination, were observed for all colonies (Supplementary Figure 3). These results indicate that the integration of the donor plasmid into pKFattB mainly occurred in the *attR* site.

**Figure 4:**
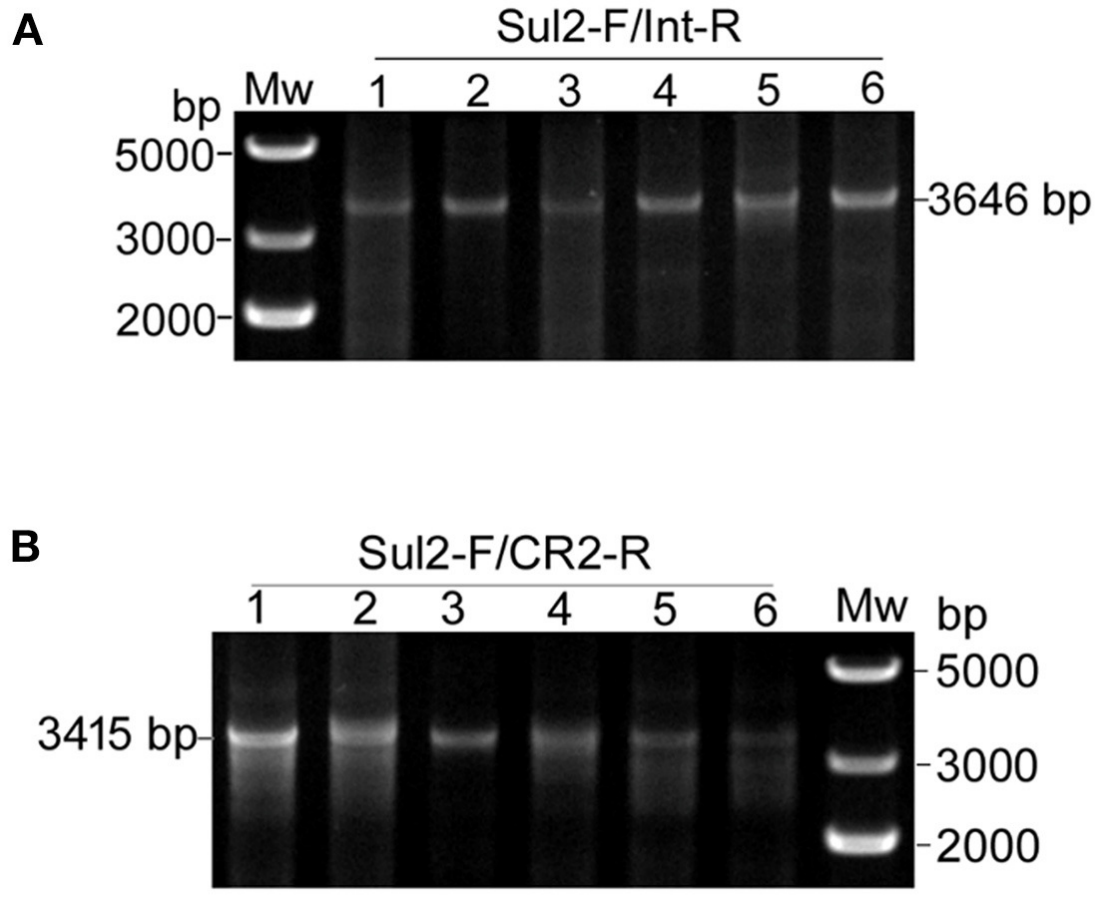
Detection of pKFattB-GItetWsul2 and pKFattB-CR2sul2 structures in recombinant colonies. PCR amplification using the primer pairs Sul2-F/Int-R and Sul2-F/CR2-R for the detection of GItetWsu2-pKFattB **(A)** and pKFattB-CR2sul2 **(B)** structures, respectively. PCR reactions were conducted on the same six recombinant colonies as in Figure 3E.

Second, we tested for the presence of structures matching the integration of the circular intermediates in the same 12 selected plasmid extracts. Primer pair Sul2-F/Int-R was used to detect pKFattB*-*GItetWsul2 structure, expected to produce an amplification size of 3,646 bp (Figure 3C). However, we only obtained bands around 10 kb which represented amplification of the donor plasmid from the cointegrates. We therefore digested the recombinant plasmids with *Nco*I restriction enzyme, which cuts only the backbone of the donor plasmid, resulting in digestion of the cointegrates. Using the *Nco*I digested products as a template, we obtain the expected 3,646 bp band for all six tested colonies (Figure 4A). Similarly, the primer pair Sul2-F/CR2-R was used for the detection of pKFattB–CR2sul2 structure after digestion of the cointegrates using *Nco*I. This PCR generated a product of the expected size (3,415 bp; Figure 4B), indicating that pKFattB-GItetWsul2 and pKFattB-CR2sul2 structures concurrently exist with cointegrate structures in the recombinant colonies.

We finally applied nanopore sequencing on the recombinant plasmids extracted from five colonies and digested them with *Xba*I, which cuts only the trap plasmid pKF*attB*, and exactly once. The total number of reads ranged from 1,382,608 to 5,033,742 (Table 1, Supplementary Table 3) for all five samples, with most of them corresponding to the original trap plasmid pKFattB (80–88%), or to regions shared by two or more recombinant structures (ambiguous regions, 4–8% of the reads), or they did not properly map on any of the structures (~10%). Nonetheless, 1,741–34,155 of the reads mapped unambiguously to the cointegrate-attR structure while only 1–225 and 5–471 reads mapped unambiguously to the cointegrate-attL and cointegrate-attLR structures, respectively. An additional 3–147 and 386–776 reads mapped unambiguously on the pKFattB-GItetWsul2 and pKFattB-CR2sul2 structures, respectively. The higher number of reads mapping to the pKFattB-CR2sul2 structure compared to other structures (except cointegrate-attR) should be treated with caution since unambiguous matches require reads long enough to map both junctions with pKFattB, which is easier to achieve for pKFattB-CR2sul2 (reads of at least 5.7-kb) than for pKFattB-GItetWsul2 (reads of at least 18-kb) or other cointegrates (reads of at least 24.2-kb). Despite this limitation, these results provided further evidence that all the five predicted structures are present, with high dominance of the cointegrate-attR structure.

**Table 1:** Number of nanopore reads unambiguously mapping on each possible recombinant structure described in Figure 3C, in five of the 12 randomly selected recombinant colonies.

**Mapped structure**	**Colony ID**				
	**I**	**II**	**III**	**IV**	**V**
Cointegrate-attL	225	1	3	5	23
Cointegrate-attLR	471	14	6	5	41
Cointegrate-attR	34,155	2,184	1,741	4,998	30,445
pKFattB-GItetWsul2	147	3	5	6	41
pKFattB-CR2sul2	460	389	776	386	686
pKFattB	1,114,689	2,528,150	1,800,859	2,147,060	4,398,420
Other reads	232,461	330,864	309,494	457,421	604,087
TOTAL	1,382,608	2,861,605	2,112,884	2,609,881	5,033,742

I–V represent the five first colonies of Figure 3E. “Other reads” include reads which mapped on regions shared by two or more structures (ambiguous reads, ~4–8% of the reads) and reads which could not map on any of the structures.

### The integrase and *att* sites of GI*sul2* are required for mobilization

To find out which genes or regions located in GI*sul2* are required for integration, several knockout plasmids were constructed from the donor plasmid pKDGItetWsul2. The deleted regions are *int, alpA/repA/C, resG, rcr2*, or the *att* sites (Supplementary Table 2). Integration of these constructs into pKFattB was tested using the same PCR detection strategy as for pKDGItetWsul2, except that the primers CRUp-R and IntUp-R replaced the primers CR2-R and Int-R, respectively, when testing for the pKFattB-CR2sul2 and pKFattB-GItetWsul2 structures because of the deletion of *rcr2 or int* in one of the constructs. The deletion of the *int* gene resulted in the complete loss of integration ability including cointegrations, GI*sul2*, and CR2-*sul2* integrations (Table 2). When the three *att* sites (*attL, attLR*, and *attR*) of pKDGItetWsul2 were deleted, only four colonies displayed the integration, but none of them carried any of the five expected recombinant structures (Table 2). A single type of integration was observed in all four clones involving the *attB* of pKFattB and a region 166 bp downstream of the deleted *attR* of pKDGItetWsul2, with the insertion site reduced to “TGGG” instead of “GAGTGGGA.” Deletion of *rep, resG*, and *rcr2* regions had no effect on the recombination efficiency and the detection of the various structures. These results indicate that the integrase of GI*sul2* and its *att* sites are required for the integration of donor plasmid, GI*sul2*, and CR2-*sul2* unit into the trap plasmid, while *rcr2*-encoded putative transposase seemingly does not have any impact on the formation of recombination structures.

**Table 2:** Effect of deleting GI*sul2*-associated regions on integrations.

**Recombinant structure**	**WT**	Δ***int***	Δ**DRs**	Δ***rep***	Δ***resG***	Δ***rcr2***
Cointegrates	+	_**−**_	${-}_{}^{*}$	+	+	+
pKFattB-GItetWsul2	+	_**−**_	_**−**_	+	+	+
pKFattB-CR2sul2	+	_**−**_	_**−**_	+	+	+

WT, wild-type (intact GItetWsul2). Δint, ΔDRs, Δrep, ΔresG, and Δrcr2 denote deleting int, attL/LR/R sites, alpA/repA/C, resG, and rcr2 in donor plasmid pKDGItetWsul2, respectively. + indicates the presence of the structure using PCR assays. ${-}_{}^{*}$Represents rare non-specific integration events. _**−**_Denotes no occurrence of integration events.

To confirm that no other factors encoded by GI*sul2* are required for integration, we constructed a minimal donor plasmid containing only the *int* gene, *attL*, and a chloramphenicol resistance determinant (*catR*) on the pKD46 backbone, designated pKDmini (6,498 bp; Supplementary Figure 4A) and tested its integration into pKFattB. After co-transformation of pKDmini and pKFattB into *E. coli* DH5α and temperature shift, transformants were plated on a medium supplemented with chloramphenicol and kanamycin, and six colonies were randomly selected. The *Xba*I restriction enzyme analysis revealed that all colonies harbored the expected 9.3-kb band corresponding to the cointegrate of pKDmini with pKFattB, and 2.8-kb band corresponding to residual pKFattB (Supplementary Figure 4B). PCR and sequencing also confirmed the result, which demonstrated that Int is sufficient for the integration through the site-specific recombination when relevant *att* sites are present.

### Unambiguous observation of GIs*ul2* and CR2-*sul2* mobilization through restriction analysis by the introduction of *sacB* into the donor plasmid and overexpression of *alpA*

**Figure 5:**
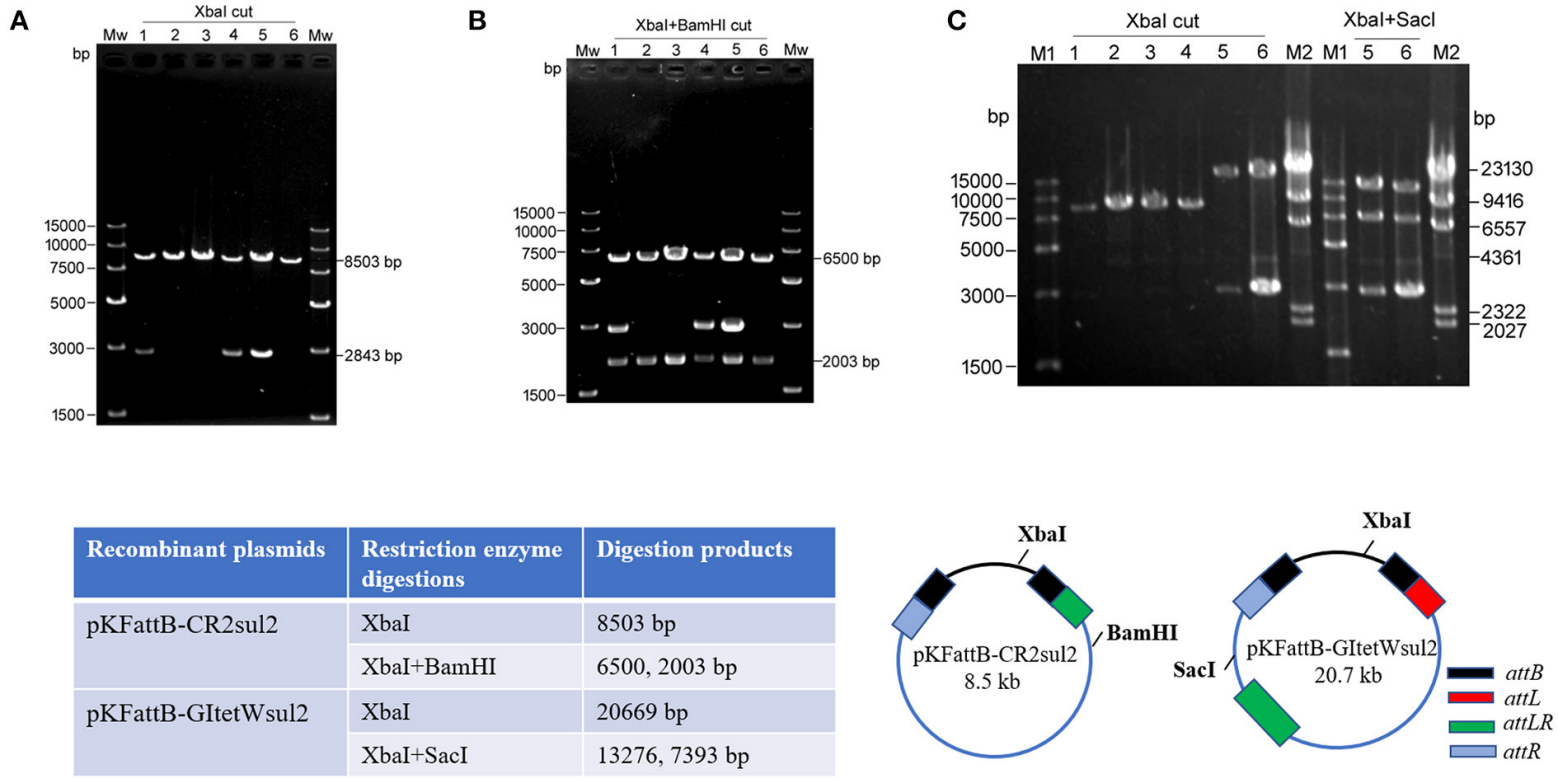
Integration of circular intermediates into the trap plasmid pKFattB under sucrose negative selection. **(A)** *Xba*I digestion of the plasmid extract from six independent recombinant colonies. Bands at 8,503 bp correspond to the recombinant plasmid pKFattB-CR2sul2 (CR2-*sul2* inserted into pKFattB) and bands at 2,843 bp correspond to original pKFattB copies. **(B)** *Xba*I and *Bam*HI double restriction of the same plasmid extracts. *Bam*HI cuts CR2-*sul2* unit once and not pKFattB, leading to the digestion of pKFattB-CR2sul2 into 6,500 and 2,003 bp bands. **(C)** Detection of GItetWsu2-pKFattB structure. Left panel, *Xba*I digestion of the plasmid extract from six independent recombinant colonies. M1, M2, different DNA markers. Right panel, *Xba*I and *Sac*I double restriction of plasmid extracts (number 5 and 6). *Sac*I cuts GI*tetWsu2* once and not pKFattB, leading to the digestion of GItetWsu2-pKFattB into 13,276 and 739 bp bands. The band at 2.8 kb is the residual pKFattB plasmid. The bottom schematics exhibit the locus of restriction enzymes and expected digestion sizes for restriction analysis.

Due to the dominance of *attR*-based cointegrate during two-plasmid mobilization experiments, the potential integration of circularized CR2-*sul2* or GI*tetWsul2* into the trap plasmid could not be observed distinctly through restriction enzyme digestions. The *sacB* gene, lethal for *E. coli* when expressed in the presence of sucrose, was therefore introduced into the backbone of pKDGItetWsul2, resulting in a new donor plasmid pKDGItetWsul2sacB (Supplementary Table 2). Cointegrates containing *sacB* are inhibited in the presence of sucrose, while colonies carrying only GI*tetWsul2* or CR2-*sul2* unit will be able to grow normally. The two-plasmid mobilization experiment was repeated using pKDGItetWsul2sacB as the donor plasmid and with the addition of 20% sucrose during the integration step, resulting in the successful growth of recombinant colonies from which 36 were randomly selected for plasmid extraction. *Xba*I digestion (cutting only pKFattB) resulted in an 8.5-kb band for all the tested colonies (6 are represented in Figure 5A), suggesting that all recombinant colonies carried the pKFattB-CR2sul2 structure. Additional digestion using the *Bam*HI enzyme (cutting CR2-*sul2* unit once) plus *Xba*I produced two bands of 6.5-kb and 2-kb, consistent with the presence of pKFattB-CR2sul2 structure (Figure 5B). These results indicate that integration of the circularized CR2-*sul2* unit into the trap plasmid can happen through site-specific recombination between the *attP*_CR2−sul2_ site and the *attB* site. No colony carrying the pKFattB-GItetWsul2 structure could have been detected using the sucrose negative selection.

AlpA is deemed to improve the excision of circular intermediates during site-specific recombination (Kirby et al., 1994; Lesic et al., 2004), and thus can increase the integration frequency of genomic islands. However, *alpA* gene deletion in our experiment did not result in significant changes for integration (Table 2). We speculated that *alpA* gene may not be expressed or maybe expressed moderately due to the lack of an efficient promoter in GI*sul2*. In view of this point, we used the expression vector pCOLADGm and constructed the resultant plasmid pCOLADalpAGm (see Section Materials and methods), under the control of a strong T7 promoter induced by IPTG. pCOLADalpAGm, pKDGItetWsul2, and pKFattB were simultaneously transformed into the *recA*-deficient strain. The integration experiment was performed on the correct strain carrying the three plasmids. Then 20 randomly selected colonies were analyzed and six samples were exhibited in agarose gel. Their extracted plasmid DNA was cut using *Xba*I and the two recombinant plasmids displayed the significant 20.7-kb band (lanes 5, 6 of left panel in Figure 5C), corresponding to the size of pKFattB-GItetWsul2. We further cut the two recombinant plasmids using double restriction enzymes *Xba*I and *Sac*I and showed the correct expected bands, 13.3 and 7.4-kb (lanes 5 and 6 of right panel in Figure 5C). *Sac*I only exists in pKFattB-GItetWsul2 structure and can cut to different sizes of fragments with *Xba*I (the bottom in Figure 5C). The weak 4,023 bp band was residual pCOLADalpAGm that was only cut by *Xba*I, and the 2,843-bp band still existed, which was the remnant pKFattB. The restriction enzyme analysis strongly indicates that GI*tetWsul2* movement occurred. Enzyme digestions of other recombinant plasmids also displayed the 8.5-kb band, indicative of CR2-*sul2* movement. These integration experiments clearly validated that GI*sul2* and CR2-*sul2* units can move into the trap plasmid.

### GI*sul2* and CR2-*sul2* units are widely distributed in proteobacteria

**Figure 6:**
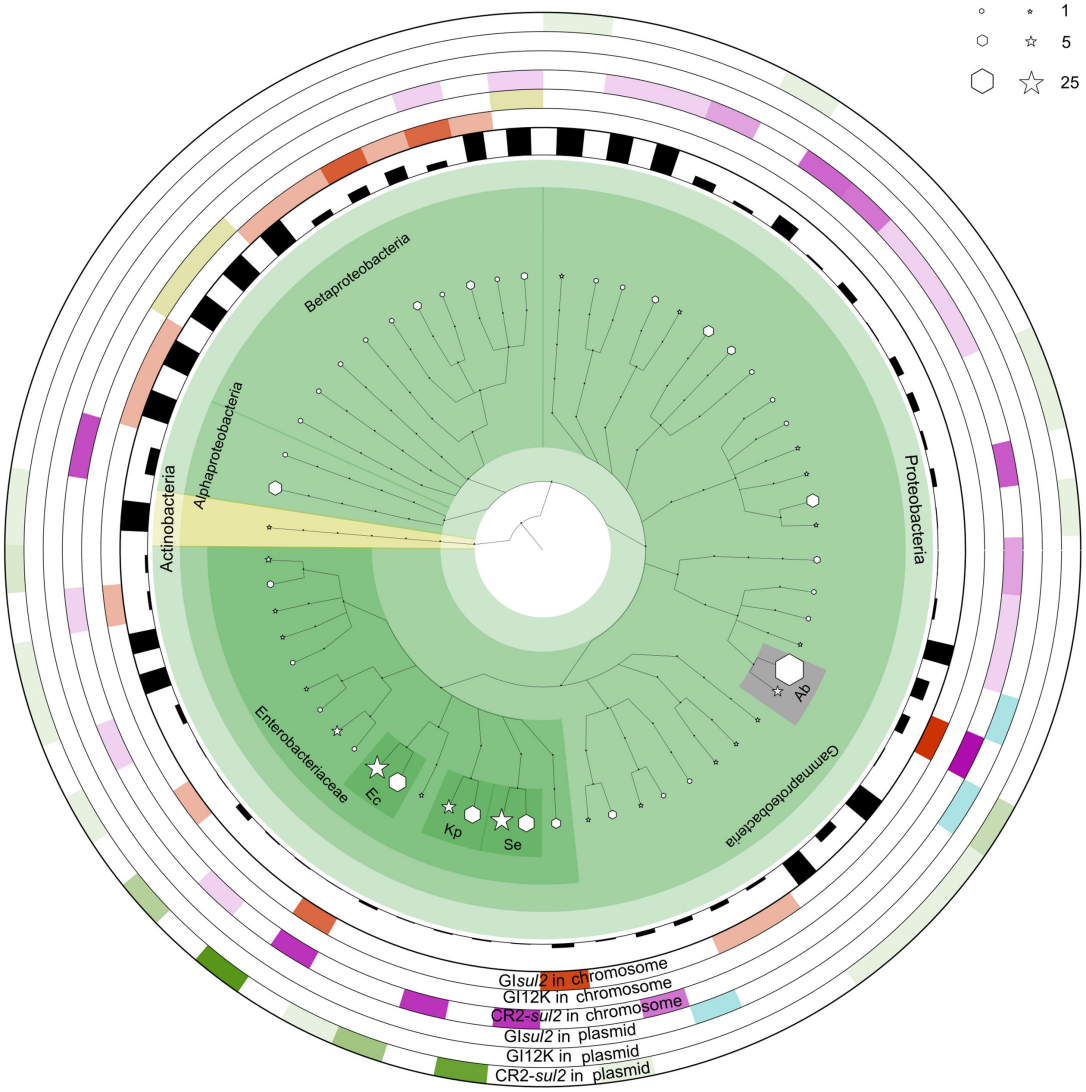
The distribution of GI*sul2*, CR2*-sul2* unit, and GI12K in chromosomes and plasmids of NCBI complete genome database. The internal species tree represent the host distribution of the three elements. The size of the hexagon and star markers denotes the number of genomes of each host carrying the corresponding elements in chromosomes or plasmids, respectively. The bar height in the inner circle represents the proportion of genomes carrying the elements among the total genome of the species in the database. The six outermost heatmaps represent each element's abundance, separated by chromosomal or plasmid localization. The color intensity corresponds to the proportion of the host in the total number of genomes carrying the given element. Orange, olive green, and purple heatmaps represent the distribution of GI*sul2*, GI12K, and the CR2*-sul2* unit in chromosomes, respectively. Blue and green heatmaps represent the dissemination of GI*sul2* and CR2*-sul2* units in the plasmids, respectively. Hosts with a total number of genomes >10 are highlighted and abbreviated as follows: Kp, *Klebsiella pneumoniae*; Ec, *Escherichia coli*; Se, *Salmonella enterica*; Ab, *Acinetobacter baumannii*.

To get a better insight into the distribution of GI*sul2*, CR2-*sul2* unit, and GI12K in bacterial species, we screened for their presence in the 169,991 complete bacterial genomes available in the NCBI GenBank database at the time of the study (see methods). The distribution of GI*sul2* shows that this element has disseminated in proteobacteria, essentially β- and γ-proteobacteria, although one instance has been detected in the α-proteobacteria *Sphingopyxis granuli* (Figure 6, Supplementary Table 4). Within γ-proteobacteria, it is restricted to two species of *Acinetobacter* (*A. baumannii* and *A. calcoaceticus*), in which eight instances were detected (6.6% of the total number of genomes for these two species), and to the *Enterobacterales* clade, in which it was sporadically found in six common pathogens (one to four instances per species, representing 0.3–33% of the species screened genomes). Within β-proteobacteria, GI*sul2* is mostly detected in species of the *Alcaliganecae* family, with eight instances distributed among five species. However, the total number of available β-proteobacterial genomes was much lower than those of γ-proteobacteria, leading to a comparatively higher prevalence of GI*sul2* in this cluster (15–100% of the species screened genomes, Supplementary Table 4). Only three of the 31 instances of GI*sul2* were detected on plasmid replicons of the complete genomes (Figure 6), consistent with its primary integration site at the 3′ end of the *guaA* gene.

CR2*-sul2* unit was detected in one Actinobacteria, one α-proteobacteria, and two β-proteobacteria, but it is widespread in γ-proteobacteria with 180 instances detected in 30 species (Figure 6). These species belong to 6 different taxonomic orders (Supplementary Table 4), including the *Aeromonadales, Oceanospirillales, Pasteurellales*, and *Vibrionales* in which no GI*sul2* was detected. The prevalence of CR2*-sul2* unit within species is also substantially higher than the prevalence of GI*sul2*; for instance, CR2*-sul2* unit was detected in 30.8, 17.8, 7.7, 6.6, and 5.4% of the *A. baumannii, Vibrio cholerae, K. pneumoniae, S. enterica*, and *E. coli* complete genomes, respectively, while GI*sul2* was detected in only 5.8, 0, 0, 0, and 0.3% of the same genomes. Contrary to GI*sul2*, CR2*-sul2* unit is also widely present on plasmids with 42% of the detections, although these plasmid-borne instances are mostly found in *Enterobacteriaceae* and *A. baumannii* (Figure 6). The last investigated element, GI12K, was found only in three complete genomes, belonging to three different species from three different orders of β-proteobacteria, and always in a chromosomal location (Figure 6).

When the NCBI draft genomes database was investigated, similar results were obtained (Supplementary Table 4). GI*sul2* was found mostly in β-proteobacteria and γ-proteobacteria with more than 10 instances detected, respectively, in *Bordetella bronchiseptica* (24% of the total genomes of the species), and *E. coli* and *A. baumannii* (0.41 and 0.97% of the total genomes). The CR2*-sul2* unit showed a wider distribution in γ-proteobacteria than GI*sul2*, but very few instances in β-proteobacteria. Its prevalence in draft genomes was similar to those in complete genomes with, e.g., 31 and 21% of the *A. baumannii* and *V. cholerae* genomes carrying it, respectively. The draft genome analysis revealed that GI12K was not restricted to β-proteobacteria since it was also detected in 19 γ-proteobacteria and one α-proteobacteria. However, its prevalence is usually extremely low in species with a sufficient number of available genomes (Supplementary Table 4). suggesting that these instances may be in fact GI*sul2* structures for which the CR2*-sul2* unit was incompletely sequenced or assembled.

## Discussion

Although several studies showed that GI*sul2* is distributed in various γ- and β-proteobacterial species (Nigro and Hall, 2011; Hamidian and Hall, 2016; Harmer et al., 2017), its mobility has never been demonstrated. In this report, we investigated the mobilization capacity of GI*sul2* integrase to perform site-specific recombination at its cognate *att* sites. Contrary to most GIs studied to date, three related *att* sites were identified in GI*sul2*: the two expected *attL* and *attR* sites at the boundaries of the element and a third, termed *attLR*, lying upstream of the *rcr2* putative transposase. This atypical GI structure carries an important mobile element, the CR2-*sul2* unit, which can also be considered as an IS*CR2*-related element that plays an important role in transferring resistance determinants (Toleman et al., 2006a). But, the mobilization mechanism of IS*CR2* is still not revealed through biological experiments. In the present study, excisions involving either *attL*-*attR* or *attLR*-*attR* complexes were both detected in RecA^+^ as well as in RecA^−^ genetic backgrounds, indicating that the integrase can excise independently the GI*sul2* and CR2-*sul2* units (bounded by *attLR* and *attR*) from their respective insertion sites. However, detection of the circular intermediates required the use of nested PCR assays, suggesting that the excision process may be rare in standard culture conditions. This phenomenon was consistent with a previous report on the excision of high-pathogenicity island in *Yersinia pseudotuberculosis* (Lesic et al., 2004).

For most GI tyrosine integrases, the recombination process requires recombination directionality factors (RDFs). Notably, excision is usually controlled by excisionase proteins such as Xis in the phage λ model (Landy, 2014). The integrase gene of GI*sul2* is followed by a gene annotated as *alpA*, which was initially proposed to act as a transcriptional regulator of CP4-57 prophage integrase, leading to the efficient excision of the prophage (Kirby et al., 1994). Later studies demonstrated that Hef, a protein homologous to AlpA, induces the excision of the high-pathogenicity island (HPI) of *Y. pseudotuberculosis* through excisionase activity (Lesic et al., 2004). Therefore, AlpA of GI*sul2* likely also acts as an excisionase as previously suggested (Harmer et al., 2017), which may not be expressed or may be expressed slightly under standard culture conditions as is the case for excisionases of other integrative elements (Guédon et al., 2017). This speculation was verified by the introduction of *alpA* gene into expression vector pCOLADGm harboring T7 promoter during IPTG induction (Figure 6). Hence, we considered that the few observed circular intermediates could have arisen from expression leakage of the *alpA* gene or occasional AlpA-empty recombinations since excisionases are mostly helper proteins (Landy, 2014; Guédon et al., 2017). While the expression of Alp increased significantly, sufficient circular intermediates were supplied, and then GI*sul2* and CR2-*sul2* mobilization can be observed easily through restriction enzyme digestions.

Our findings are not consistent with the results of Harmer et al. (2017), who did not detect GI*sul2* circular intermediates in their *E. coli* experimental strain despite a nested PCR procedure similar to ours. In their experiment, GI*sul2* was not located in its typical integration site downstream of the *guaA* gene, but in an IncC-type plasmid in which the *attL* and *attR* sites were not conserved. We suspect that this unusual insertion site hampers the integrase-mediated excision because we showed that *att* sites are essential for proper integrase-mediated recombination. Despite its preferential chromosomal integration at the 3′-end of *guaA*, GI*sul2* has been sporadically detected on plasmids at unrelated insertion sites (8, 9, 12, Figure 6). Here, we showed that elements devoid of *att* sites could still integrate into the donor plasmid, but at a very low rate and in a recombination site with reduced specificity (“TGGG” instead of “GAGTGGGA”). This suggests that Int behaves like any other integrase, for which target specificity may be loosened when no proper integration site is present in the cell (Guédon et al., 2017). However, non-specific integrations are likely a dead-end for the dissemination of GI*sul2* because of the lack of proper excision when full-length *attL/R* sites are not present. GI*sul2* is more probably an integrative and mobilizable element (IME), which relies on a helper conjugative element to transfer between bacteria (Guédon et al., 2017). The presence of plasmid-related transfer proteins into the element strongly supports this hypothesis, and the reported amino-acid similarity of these proteins to those found in IncP plasmids suggests that members of this plasmid family would be the helper elements (Harmer et al., 2017).

Finally, we showed that when the dominant cointegrate recombinants are eliminated by sucrose negative selection, integration of circularized intermediates into *attB* can be detected. Notably, integration of GI*sul2* circular intermediate into the *attB* site of trap plasmid was observed through restriction enzyme analysis in *recA*^−^ genetic context, indicating that the integrase can perform the final step of GI*sul2* mobilization when molecular substrates are available. Even more interesting is our detection of CR2-*sul2* integration in the *attB* site of the trap plasmid. CR2 transposase is very often located in the vicinity of *sul2* with various ARGs in between, and several genomic evidences suggest that they represent independent mobile or mobilizable units (Leclercq et al., 2016; Xu et al., 2017). These CR2-*sul2* units are therefore recognized as vectors of dissemination of antibiotic resistance in environmental bacteria and important clinical pathogens such as *Vibrio* spp., *Pseudoalteromonas* spp., *Salmonella enterica, S. flexneri*, and *A. baumannii* (Beaber et al., 2002; Toleman et al., 2006a; Yau et al., 2010; Leclercq et al., 2016; Harmer et al., 2017; Xu et al., 2017; Shi et al., 2019; Wüthrich et al., 2019). Especially, plasmid-borne IS*CR2* carrying the high-level tigecycline resistance gene *tet(X)* has emerged in *E. coli* (Fang et al., 2019; He et al., 2019; Sun et al., 2019), which hints that we should pay close attention to the rise of this element. Interestingly, the boundaries of these transposons have been predicted to lie 119 bp upstream of the *rcr* gene and 304 bp upstream of *sul2* (Leclercq et al., 2016), which correspond to the middle of the core recombination region of *attLR*, and the 3′-end of *attR*, respectively. In another study conducted on *Aeromonas caviae*, two independent ARG-carrying CR2*-sul2* units are inserted in the *attR* core recombination region of a GI inserted in *guaA*. The GI harbors an integrase related to that of GI*sul2* (Shi et al., 2019). Both integrations recreated perfect *att* core recombination regions (Shi et al., 2019), and very likely functional *att* sites. These observations, together with the results presented here, suggest that the integrase of GI*sul2*, or other integrases of the same family, may be the main drivers of the wide dissemination of CR2-*sul2* units among proteobacteria. Future studies are need further to deeply investigate the influence of AlpA and possible host factors on integration or excision, providing a comprehensive understanding of mobilization.

## Materials and methods

### Bacterial strains and culture conditions

The genomic DNA of *S. flexneri* 51575 (kindly provided by Prof. Jianguo Xu from Chinese CDC) was extracted with the TIANamp Bacteria DNA Kit (TIANGEN BIOTECH, Beijing) and used to amplify the complete sequence region of GI*sul2*. *E. coli* DH5α deficient in *recA* was used for plasmid cloning and to perform all the integration experiments. IN-5 (KU736870) and IN-11 (KU736876) are, respectively *tetW*-containing and CR2-containing genomic regions cloned from pig manure's uncultured bacteria (Leclercq et al., 2016) and used for plasmid constructions. Unless otherwise stated, all strains were cultured at 37°C in a liquid LB medium. All plasmid constructions mentioned in this study are detailed in Supplementary Table 2.

### PCR detection of circular intermediates

Detection of circular intermediates was performed using a nested PCR strategy on crude DNA from boiled overnight cultures (Lesic et al., 2004). To determine GI*sul2* circular intermediates, the primer pair F1/R1 was first used to amplify the junction. Then the Sul2-F/Int-R primer pair was used for the second round of PCR amplification using the first-round PCR product as the template. Similarly, to detect CR2-*sul2* unit and GI12K circular intermediates, a couple of primer pairs F1/R2 then Sul2-F/CR2-R, and tru-F1/R1 then tru-F2/Int-R were used, respectively. PCR conditions were as follows for both rounds: pre-denaturation at 95°C for 5 min; 30 cycles of denaturation at 94°C for 30 s, annealing at 55°C for 30 s, extension at 72°C for 1 min; and then a final extension at 72°C for 5 min. All PCR amplifications were performed using the ploymerase TransStart^®^ FastPfu Fly DNA Polymerase, which is a hot start, ultra-high fidelity, and high-processivity DNA polymerase (TRANS, Beijing, China). These PCR amplicons could also have theoretically arisen from chromosomal tandem duplications of each of the elements, but the fact that a nested PCR procedure was necessary to produce visible bands, together with the absence of such duplication in the genome sequence of the used strain, makes this possibility highly unlikely.

### Construction of a two-plasmid assay system

The donor plasmid pKDGItetWsul2 was constructed in a two-step process. The first step consisted of the construction of a CR2′-*tetW*-sul′ fragment containing a constitutively expressed *tetW* gene. The *tetW* gene (2,070 bp) was amplified from the DNA of the IN-05 clone by using tetW-F and tetW-R primers. The PCR product was digested together with plasmid pUC19 by *Pst*I and *Eco*RI enzymes and ligated using a T4 DNA ligase (New England Biolabs, Ipswich, MA, USA). The plasmid was termed pUC-*tetW*. The 3′ end of CR2 (1,156 bp) harboring the putative *ori* end was amplified from the IN-11 clone using G-UNIT-F/Rev-Pstv28cr2 primers. The *tetW* plus the P_lacZ_ promoter sequences (2,403 bp) were amplified from pUC-tetW using G-tetWF/Rev-tetW primers. The partial *sul2* sequence (789 bp) was amplified from IN-11 using Fwd-Pstv28cr2/G-UNIT-R primers. The above three fragments were assembled using the NEBuilder kit to obtain the CR2′-*tetW*-sul′ fragment (4,313 bp). The second step consisted of the aggregation of the CR2′-*tetW*-sul′ fragment with GI*sul2* in a pKD46 backbone, lacking *gam, bet*, and *exo* regions, from four genomic fragments. First, the pKD46 backbone segment (4,199 bp) was obtained by PCR amplification using G-PKD-F and G-PKD-R primers from an intact pKD46 template. Second, the G-UP-floRF11 and G-UP-R primers were used to amplify a 2,505 bp fragment including the florfenicol resistance gene *floR* and the 5′ end of CR2 bearing *attLR* using the genomic DNA of the IN-11 clone as the template. The third fragment was the CR2′-*tetW*-sul′ constructed above. The fourth fragment included the 5′ end of *sul2* and its upstream sequence (1,005 bp), obtained using G-DOWN-F and G-DOWN-R11 primers with DNA of the IN-11 clone as the template. The above four fragments were assembled using NEBuilder HiFi DNA Assembly Cloning Kit, resulting in the plasmid pKD-tetW-CR2. The 12-kb sequence of GI*sul2* upstream of the CR2-*sul2* unit was amplified from the genomic DNA of *S. flexneri* 51575 using 15K-F/CRUp-R primers, resulting in a 12,256 bp fragment. The recombinant plasmid pKD-tetW-CR2 was digested by BbvC1 restriction enzyme, producing overlap sequences with the corresponding two terminal regions of the GI*sul2* 12-kb fragment. The two fragments were ligated using the NEBuilder HiFi DNA Assembly Cloning Kit, resulting in the donor plasmid pKDGItetWsul2.

To construct the trap plasmid pKFattB, the *attB* region (811 bp), including the *attB* site (19 bp) and its upstream (319 bp) and downstream (473 bp) regions, was amplified from the chromosome of *E. coli* DH5α with primers G-GMP-SF and G-GMP-SR. These *attB* upstream and downstream regions corresponded to the 3′ end of *guaA* (336 bp) and the 5′ end of a hypothetical protein (381 bp), respectively. The high copy plasmid pKF18k-2 (Takara Bio Inc., Kasatsu, Japan) was digested using *Xba*I/*Hind*III enzymes and the digestion product was assembled together with the amplified *attB* region using NEBuilder HiFi DNA Assembly Cloning Kit (New England Biolabs, Ipswich, MA, USA), resulting in the trap plasmid pKFattB.

### Chromosomal integration and two-plasmid mobilization experiment in *recA*-deficient *E. coli*

pKDGItetWsul2 was transformed into *E. coli* DH5α, which was cultured on LB agar plates containing ampicillin (100 μg/ml) at 30°C. Single colonies (transformants) were selected and cultured at 30°C for 24 h in liquid medium containing tetracycline (20 μg/ml), then the temperature was increased to 42°C for 16 h to stop pKDGItetWsul2 replication. The resulting cultures were plated on LB medium containing tetracycline (20 μg/ml) at 42°C and then 12 colonies were selected and cultured in liquid LB at 42°C overnight. Crude DNA from these recombinant colonies was used as the template for PCR amplification.

The two-plasmid mobilization assay was performed according to a previous report (Wilde et al., 2008). pKDGItetWsul2 and pKFattB were co-transformed into *E. coli* DH5α, which was cultured on LB agar plates containing ampicillin (100 μg/ml) and kanamycin (50 μg/ml) at 30°C. The integration experiment was performed as in the chromosomal integration experiment, except that 50 μg/ml kanamycin was added to each medium. A total of 12 recombinant colonies were selected and their plasmids were extracted using TIANprep Mini Plasmid Kit (TIANGEN BIOTECH, Beijing). These recombinant plasmids were used as a template for PCR amplification and enzyme restriction analysis.

### Nanopore sequencing and analysis

Five of the twelve recombinant plasmid extracts were digested with the *Xba*I restriction enzyme, cutting specifically the trap plasmid pKF*attB* 324 bp upstream of the *attB* site. The digestion products were sequenced using the PromethION platform (NextOmics, Wuhan, China). The raw reads data were demultiplexed and filtered with the Porechop v0.2.4 software (Wick, 2018). The quality control values were set as follows: (mean_qscore_template) ≥7 and sequence length ≥1,000 bp. Residual barcoding adapters were removed by trimming 100 bp on both sides of each read using cutadapt v2.9 (Martin, 2011). The filtered high-quality reads in fasta format were mapped against the pKF*attB* sequence and the sequence of each of the five expected recombinant structures using graphmap2 v0.6.3 (Sović et al., 2016) with the end-to-end owler parameter. The number of input structures mapped by each read was evaluated, and reads were categorized as unambiguous when they were properly mapped on only one structure and ambiguous when they were properly mapped on two or more structures.

### Deletion of the *int* gene, *att* sites, and other mobility-associated genes or regions in GI*sul2*

Using pKDGItetWsul2 as a template, an *int*-deficient fragment of GI*sul2* (*int* completely deleted) was amplified using the delINT-F/R primers. The backbone of pKDGItetWsul2 segment including the temperature-sensitive replicon and the ampicillin resistance marker was obtained using primers V15K-F/R. The two fragments were assembled using the NEBuilder Assembly Tool to produce the pKDGItetWsul2-Δint donor. Using the same strategy, the pKDGItetWsul2*-*Δ*rcr2*, pKDGItetWsul2-Δ*resG*, and pKDGItetWsul2-Δ*rep* donor plasmids were constructed using the delCR2-F/R and V15K-F/R, del-ser-F/R and Res-F/R, del-repF/R and V15K-F/V-D-repR primer pairs, respectively. The *att*-depleted plasmid donor was constructed as follows: *attL*-/*attR*-deficient fragment and the vector backbone fragment were amplified by PCR using del-21bp-F/R and VPKD-F/R primers, respectively, with pKDGItetWsul2 as the template. The two fragments were assembled using NEBuilder Assembly Tool, resulting in the *attL/R*-deficient plasmid. D-all21bpF/R and V-D-all21F/R primer pairs were then used to amplify two fragments devoid of *attLR* from the *attL/R*-deficient plasmid. The latter two fragments were assembled using NEBuilder Assembly Tool to construct the final *attL/LR/R*-deficient plasmid, named pKDGItetWsul2-Δatt. All primer sequences were designed according to the manufacturer's instructions (http://nebuilder.neb.com/), as shown in Supplementary Table 1. The constructed deletion plasmids served as donor plasmids for integration experiments using the two-plasmid mobilization system as described above.

### Construction of pKDGItetWsul2sacB and pCOLADGm, and the mobilization assay

The *Mlu*I restriction enzyme (NEB) was used to cut pKDGItetWsul2 (24157 bp) and the *sacB* original vector pDM4 (7,104 bp). *Mlu*I cuts the araBAD promoter within pKDGItetWsul2 and cuts pDM4 twice, creating a 3.8-kb fragment that contains a chloramphenicol resistance gene and the *sacB* gene plus its promoter. The two fragments were purified and then ligated using a T4 DNA ligase (NEB). The ligation product was transformed into *E. coli* DH5α and cultured in LB containing tetracycline and chloramphenicol. Transformants were verified by *Mlu*I restriction analysis and sequencing. The new donor plasmid containing *sacB* was designated pKDGItetWsul2sacB and used for integration experiments with the trap plasmid pKFattB, as well as for the chromosomal integration experiment. Sucrose (20%) was used in addition to tetracycline and kanamycin in the LB medium used for the selection of the recombinant colonies.

To introduce *alpA* encoding region into the new two-plasmid system composed of pKDGItetWsul2sacB and pKFattB, pCOLADuet-1 (ColA origin, different from the origins of two-plasmid system) was used for overexpression of *alpA* gene under the control of IPTG. pCOLADuet-1 carries the kanamycin resistance determinant, similar to pKFattB. We used the gentamycin resistance gene derived from pEX18Gm to replace the kanamycin resistance gene. BspHI cut pCOLADuet-1 and pEX18Gm, respectively. Then using NEB T4 ligase ligated the two parts and then formed pCOLADGm. Amplification of *alpA* with primers alpANcoI-F and alpASalI-R was performed using the donor plasmid as the template. The PCR product was digested with NcoI and SalI restriction enzymes. The same double enzymes cut pCOLADGm. The two fragments were ligated by using T4 ligase. The resultant plasmid pCOLADalpAGm was constructed successfully through enzyme digestion and sequencing verification. pCOLADalpAGm, pKDGItetWsul2, and pKFattB were simultaneously transformed into the *recA*-deficient strain DH5α, and the LB agar plate containing tetracycline (10 μg/ml), kanamycin (50 μg/ml), and gentamycin (10 μg/ml) was screened at 30°C for 48 h. The correct strain was incubated in LB liquid culture carrying 20% sucrose, tetracycline (10 μg/ml), kanamycin (50 μg/ml), gentamycin (10 μg/ml), and IPTG (0.5 mM) at 30°C for 24 h. The culture dilution was plated LB harboring the same components as the liquid culture at 37°C for 48 h. Plasmids of the colonies extracted were the template, which was used for restriction enzyme analysis.

### Bioinformatics methods and the distribution analysis of GI12K, GI*sul2*, and CR2-*sul2*

A total of 169,991 bacterial genomes were downloaded from the NCBI genome database [ftp://ftp.ncbi.nlm.nih.gov/genomes/genbank/] on 15 November 2018. The taxonomic lineage of each genome was retrieved from the NCBI taxonomy database. First, a search of regions matching the Int sequence with 95% amino acid identity and 98% coverage was performed. Preliminary results on the complete genomes database showed that the GI12K sequence coverage systematically exceeded 70% in genomes where a match on Int was detected with these parameters (Supplementary Figure 5). Hence, genomes returning a match to Int with 95% amino acid identity and 98% coverage were considered to carry GI12K. A search of the *rcr2* and *sul2* genes with a BLASTN cutoff of 95% nucleotide similarity was performed independently on all genomes, and genomes including both genes were considered as carrying the CR2*-sul2* element. Preliminary results on complete genomes indicated that it is indeed the case since all the CR2*-sul2* regions detected this way were surrounded by the typical 119 and 304 bp conserved regions ending at the core recombination sites of the *att* loci (Leclercq et al., 2016), despite inner structural variation. The distribution of the GI12K, GI*sul2*, and the CR2-*sul2* unit was then estimated as follows: when only the match to the integrase was satisfied, the genome was considered to carry GI12K; when only the match to *rcr2* and *sul2* regions was satisfied, the genome was considered to carry a CR2*-sul2* unit; finally, when all matches were satisfied, the genome was considered to carry GI*sul2*. This analysis was performed on the complete genome database and the draft genome database independently. The chromosomal or plasmid localization of the detected elements was returned only for the complete genome database, where the replicon type is known. Then, to illustrate the distribution of GI12K, GI*sul2*, and CR2-*sul2*, the bacterial taxonomic framework of the 169,991 bacterial genomes was built by using the software GraPhlAn based on the taxonomic lineage information (Asnicar et al., 2015), with the quantities for each GI being mapped to the taxonomic framework.

## Data availability statement

The datasets presented in this study can be found in online repositories. The names of the repository/repositories and accession number(s) can be found at: https://www.ncbi.nlm.nih.gov/genbank/, PRJNA608816.

## Author contributions

GZ and QC designed and executed the work. GZ wrote the manuscript. JF and SL revised substantially the manuscript. JL and RG performed some detailed experiments. LD, NT, and FZ performed the distribution and drawn the relevant figure. YS and SL performed the bioinformatic analysis. CW did some figures. All authors contributed to the article and approved the submitted version.

## Funding

This work was supported by the National Natural Science Foundation of China (grants 31870134, 32070075, 81861138053, and 31872632).

## Conflict of interest

The authors declare that the research was conducted in the absence of any commercial or financial relationships that could be construed as a potential conflict of interest.

## Publisher's note

All claims expressed in this article are solely those of the authors and do not necessarily represent those of their affiliated organizations, or those of the publisher, the editors and the reviewers. Any product that may be evaluated in this article, or claim that may be made by its manufacturer, is not guaranteed or endorsed by the publisher.
