# Supplementary material for: The integrase of genomic island GIsul2 mediates the mobilization of GIsul2 and ISCR-related element CR2-sul2 unit through site-specific recombination
Source: Front Microbiol. 2022 Aug 1;13:905865. doi: 10.3389/fmicb.2022.905865 (PMC9376610; doi:10.3389/fmicb.2022.905865)
Supplement: Supplementary file 1 [file Table_1.DOCX]

Table S1 Primers designed for this study.

|  | Name | Sequence (5’-3’) |
| --- | --- | --- |
| Detection of excised circular intermediates: | | |
|  | F1 | gcgtcgggattgctggat |
|  | R1 | catccgagtcgtaccgtgat |
|  | Int-R | ccgaccgttgattgagtagttgt |
|  | Sul2-F | gagaaactgtccgaggttatg |
|  | R2 | ttccctgctctgccataagc |
|  | CR2-R | cgtcaacgatctgatagagaag |
|  | tru-F1 | atgctttttcgcctggccttg |
|  | tru-F2 | catgaagaagccgaaccaagac |
| Detection of cointegrates structures: | | |
|  | pKF-F | cacctctgacttgagcgtcg |
|  | pKF-R | tgctgcaaggcgattaagttg |
|  | GMP-F | gaagtgaagaaagagtactgtgac |
|  | pKD-F2 | accttggtgtcctgctatttga |
|  | M13-47 | cgccagggttttcccagtcacgac |
|  | Res-F | ttcgtggaggacgcattgac |
|  | pKD-R | gatctcaatggttcgttctc |
|  | GMP-R | cgaatttatcgatcaggaact |
|  | CRUp-R | cttagcggaaagttcttttaccc |
|  | IntUp-R | cttacgaaacagtaacttatagaa |
| Construction of the donor plasmid pKDGItetWsul2: | | |
|  | tetW-F | acgcctgcaggtaagccgtcatacttctgtttgt |
|  | tetW-R | acgcgaattccgccttctccgcactca |
|  | G-UNIT-F | ctgtttctcgacggtgtgtatgtcgagcaatc |
|  | Rev-Pstv28cr2 | acgtataggaagaataaacgccc |
|  | G-tetWF | cgtttattcttcctatacgtctttgagtgagctgataccg |
|  | Rev-tetW | ggcccgctgccgacattacgccagtatgtatctttac |
|  | Fwd-Pstv28cr2 | tgtcggcagcgggccaaa |
|  | G-UNIT-R | gaaatcatctgccaaactcgtcgttatgcattc |
|  | G-PKD-F | ccatgggtatggacagttttc |
|  | G-PKD-R | agtattaatatccattttttataacctccttag |
|  | G-UP-floRF11 | gttataaaaaatggatattaatactgtgcgcccatgccgcattg |
|  | G-UP-R | tcgacatacacaccgtcgagaaacagcatgtg |
|  | G-DOWN-F | taacgacgagtttggcagatgatttcgccaatt |
|  | G-DOWN-R11 | gggaaaactgtccatacccatggctaaccatatacgcgcaaacataag |
|  | 15K-F | tgtctagcaacggcaggcatttcggctgaggtaccattgagtgggaatgattcc |
| Construction of the recipient plasmid pKFattB: | | |
|  | G-GMP-SF | tttgctggccttttgctcatctagagaagtgaagaaagagtactgtgac |
|  | G-GMP-SR | ttgtaaaacgacggccagtaagcttgaaatccagcaagagaagca |
| Construction of the KO donor plasmids: | | |
|  | delINT-F | tcggagacgaagaaggacaaggtacttcgggaggcagtaagggttga |
|  | delINT-R | gtatcgttggccttcaggctcg |
|  | V15K-F | cgagcctgaaggccaacgatac |
|  | V15K-R | gtaccttgtccttcttcgtctccg |
|  | delCR2-F | cggagacgaagaaggacaaggtac |
|  | delCR2-R | ttggtatcgttggccttcaggctcggttccgcgtccttgcaatactg |
|  | del-ser-F | tcggagacgaagaaggacaaggtac |
|  | del-ser-R | gctcgtcgtcttggttcggcttcttcatacttgcgggctcctttgggc |
|  | Res-R | gtaccttgtccttcttcgtc |
|  | del-repF | gacttcgggaggcagtaagggttgacgctccccttctgccgtcgtaa |
|  | del-repR | gtatcgttggccttcaggctcg |
|  | V-D-repR | tcaacccttactgcctcccga a |
|  | del-21bp-F | atataaaagataaaaatgtcttgttcgaatcagcggtagaacaaggtttaac |
|  | del-21bp-R | aggtgcctggctgtgccagacgcggcctcagccgaaatgcctgcc |
|  | VPKD-F | ccg cgtctggcacagcca |
|  | VPKD-R | aacaagacatttttatcttttatattcaatggc |
|  | D-all21bpF | tcacgcagtgaagaccgcggagtgaccgcgtctggcacagcca |
|  | D-all21bpR | ttgctagacattgccagccagtgccaacaagcacacaaaaccctttagtgacaac |
|  | V-D-all21F | ggcactggctggcaatgtc |
|  | V-D-all21R | tcactccgcggtcttcac |
| Amplification of *alpA* gene from pKDGItetWsul2 | | |
|  | alpANcoI-F | gatccatggccatgcaaaatataaagaccc |
|  | alpASalI-R | gatgtcgaccataagatcaagagccatataac |
