# Supplementary material for: The integrase of genomic island GIsul2 mediates the mobilization of GIsul2 and ISCR-related element CR2-sul2 unit through site-specific recombination
Source: Front Microbiol. 2022 Aug 1;13:905865. doi: 10.3389/fmicb.2022.905865 (PMC9376610; doi:10.3389/fmicb.2022.905865)
Supplement: Supplementary file 2 [file Table_2.DOCX]

Table S2 Plasmid constructs used in this study.

| Plasmid name | Description | Reference |
| --- | --- | --- |
| pKF18k-2 | pUC type vector, ColE1, high copy plasmid, kanamycin resistance gene (*kanR*) | Takara |
| pKFattB | Insertion of the *attB* region of GI*sul2* into pKF18k-2 (*kanR*) | This study |
| pKD46 | Temperature sensitive replication, oriR101, low copy plasmid, ampicillin resistance gene (*ampR*) | Datsenko and Wanner 2000 |
| pDM4 | A plasmid carrying *sacB* and a chloramphenicol resistance gene, (*catR*) | This study |
| pKDGItetWsul2 | Insertion of a modified GI*sul2* sequence (replacement of △*glmM* with *tetW*) into pKD46 backbone (*ampR,tetR)* | This study |
| pKDtetWGIsul2-Δ*int* | Deletion of *int* of pKDtetWGIsul2 *(ampR,tetR*) | This study |
| pKDtetWGIsul2-Δatt | Deletion of all three *att* sites (*attL/LR/R*) of pKDtetWGIsul2 *(ampR,tetR*) | This study |
| pKDtetWGIsul2-Δ*resG* | Deletion of *resG* of pKDtetWGIsul2 *(amp^R^,tet^R^*) | This study |
| pKDtetWGIsul2-Δ*rep* | Deletion of the *rep* region (*repA/C* and *alpA)* of pKDtetWGIsul2 *(ampR,tetR*) | This study |
| pKDGIsul2sacB | Insertion of *sacB* gene plus its promoter and the chloramphenicol resistance gene into pKDGItetWsul2 *(catR, amp*R*,tetR*) | This study |
| pEX18Gm | Gentamycin resistance marker, pUC18-derived vector | Hoang et al. 1998 |
| pCOLADuet-1 | Expression vector bearing T7 promoter, kanR | Novagen |
| pCOLADGm | Insertion of gentamycin resistance marker from pEX18Gm into pCOLADuet-1, gmR | This study |
| pCOLADalpAGm | Introduction of *alpA* gene to pCOLADGm, gmR | This study |
