# Supplementary material for: The integrase of genomic island GIsul2 mediates the mobilization of GIsul2 and ISCR-related element CR2-sul2 unit through site-specific recombination
Source: Front Microbiol. 2022 Aug 1;13:905865. doi: 10.3389/fmicb.2022.905865 (PMC9376610; doi:10.3389/fmicb.2022.905865)
Supplement: Supplementary file 3 [file Table_3.DOCX]

| Sample | Type | Bases (bp) | Reads number | Reads mean length (bp) | Reads N50 (bp) | Longest Reads (bp) |
| --- | --- | --- | --- | --- | --- | --- |
| I | Raw Reads | 5,474,246,055 | 1,545,867 | 3,541.21 | 2,933 | 117,930 |
|  | Filtered Reads | 4,886,849,508 | 1,382,608 | 3,534.52 | 2,928 | 73,118 |
| II | Raw Reads | 8,205,152,864 | 2,997,534 | 2,737.30 | 2,884 | 614,836 |
|  | Filtered Reads | 7,842,990,335 | 2,861,605 | 2,740.77 | 2,886 | 55,828 |
| III | Raw Reads | 5,934,077,836 | 2,226,008 | 2,665.79 | 2,876 | 303,112 |
|  | Filtered Reads | 5,638,787,670 | 2,112,884 | 2,668.76 | 2,878 | 56,966 |
| IV | Raw Reads | 7,210,751,307 | 2,732,143 | 2,639.23 | 2,887 | 331,012 |
|  | Filtered Reads | 6,887,913,036 | 2,609,881 | 2,639.17 | 2,889 | 75,613 |
| V | Raw Reads | 15,458,119,315 | 5,305,025 | 2,913.86 | 2,902 | 756,474 |
|  | Filtered Reads | 14,661,725,078 | 5,033,742 | 2,912.69 | 2,904 | 70,052 |

Table S3 Nanopore sequencing data for the five samples
