# Supplementary figures and images for: The integrase of genomic island GIsul2 mediates the mobilization of GIsul2 and ISCR-related element CR2-sul2 unit through site-specific recombination
Source: Front Microbiol. 2022 Aug 1;13:905865. doi: 10.3389/fmicb.2022.905865 (PMC9376610; doi:10.3389/fmicb.2022.905865)

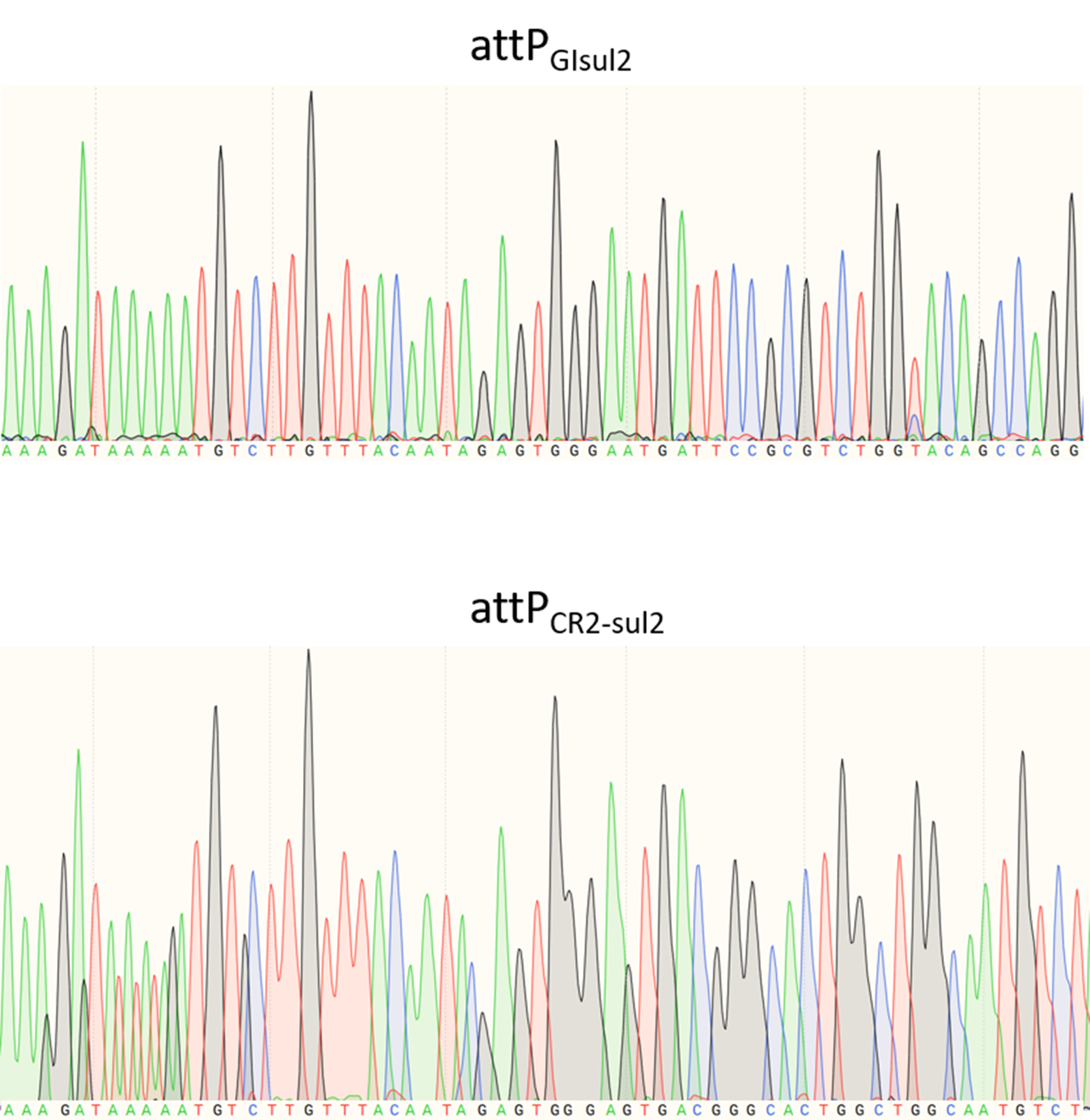

Supplement: Supplementary file 5 [file Image_1.TIF]

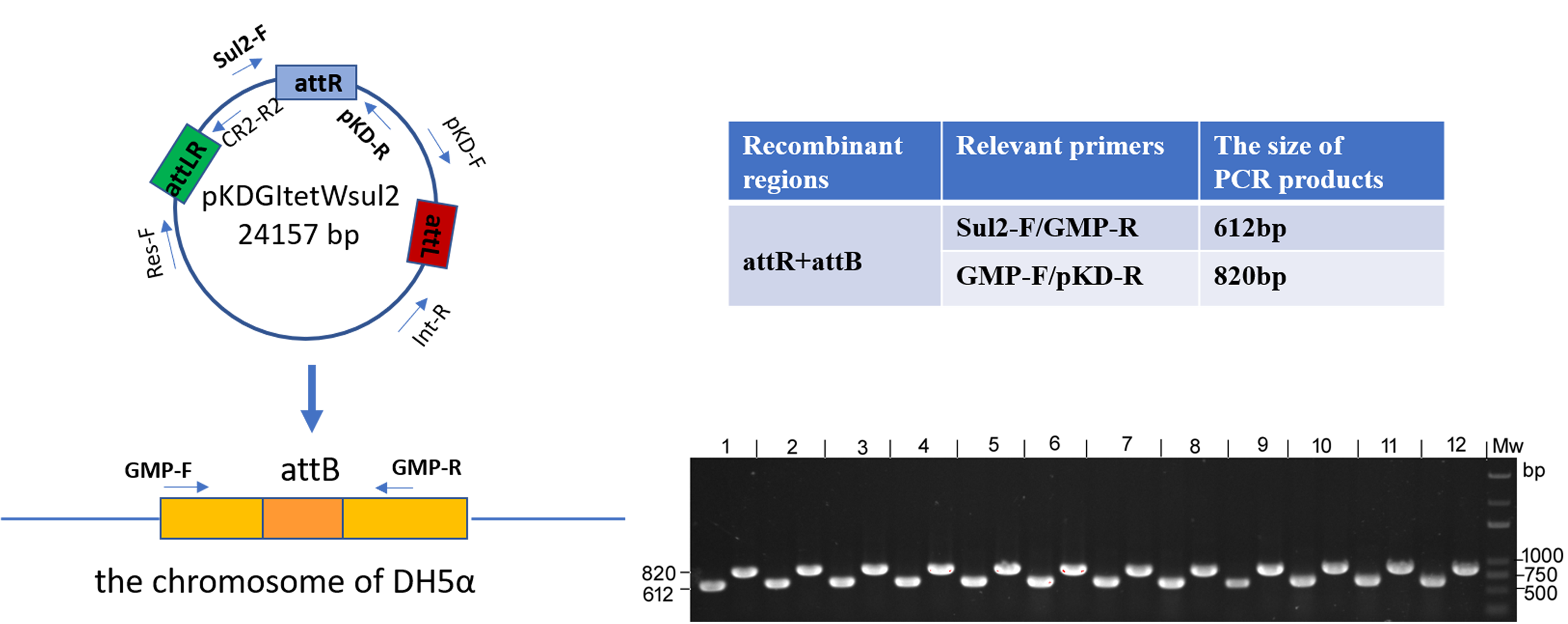

Supplement: Supplementary file 6 [file Image_2.TIF]

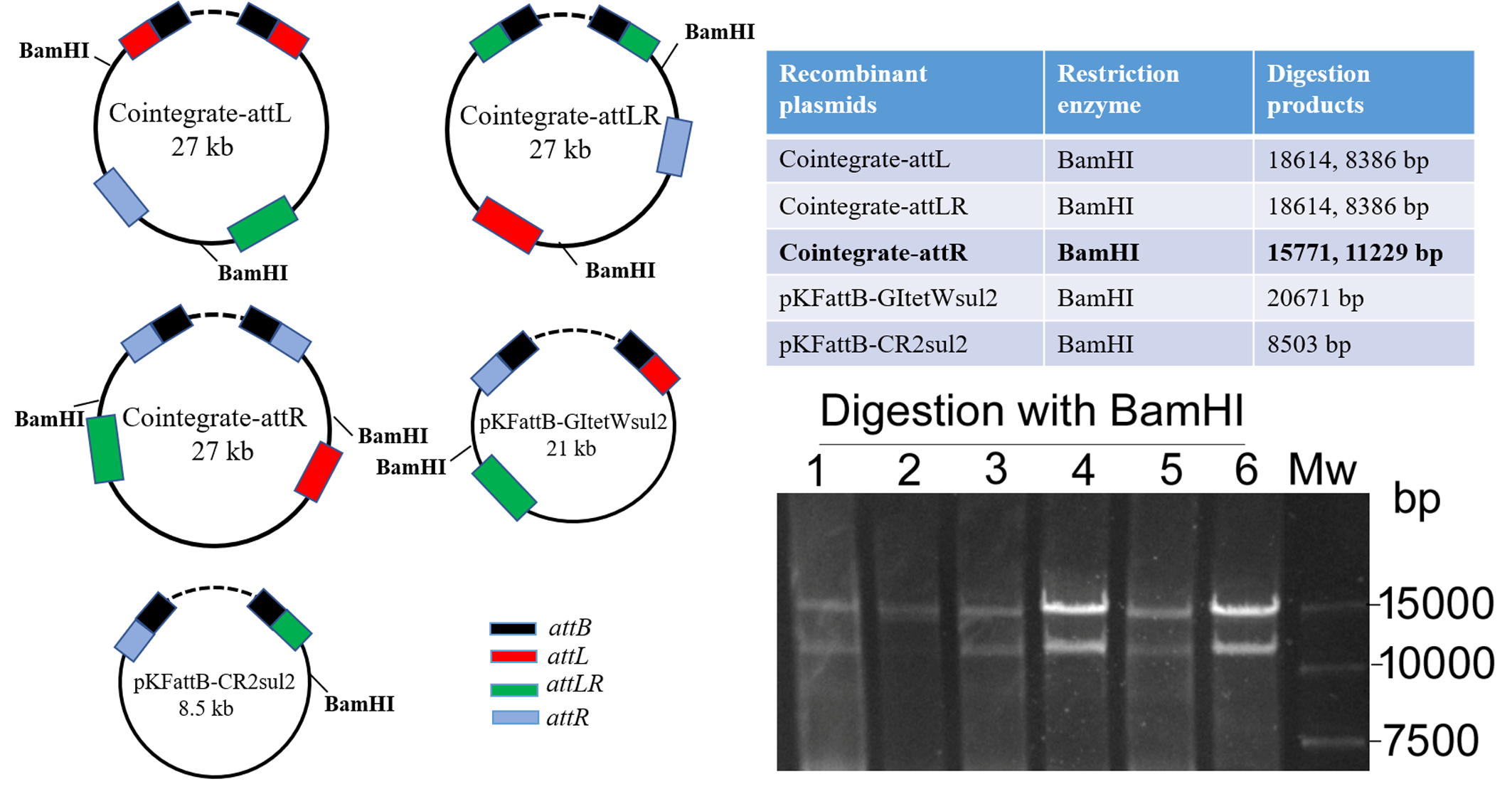

Supplement: Supplementary file 7 [file Image_3.TIF]

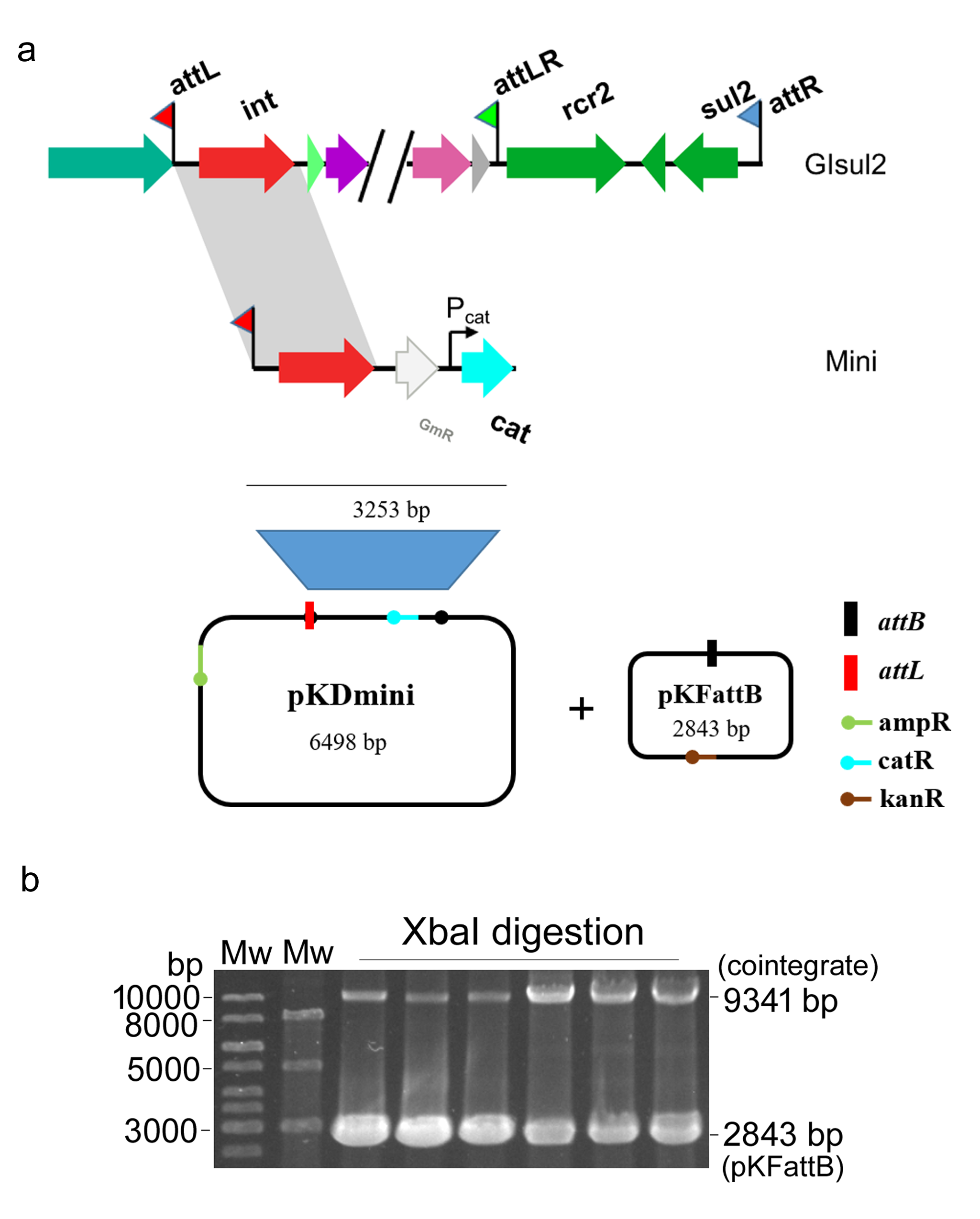

Supplement: Supplementary file 8 [file Image_4.TIF]

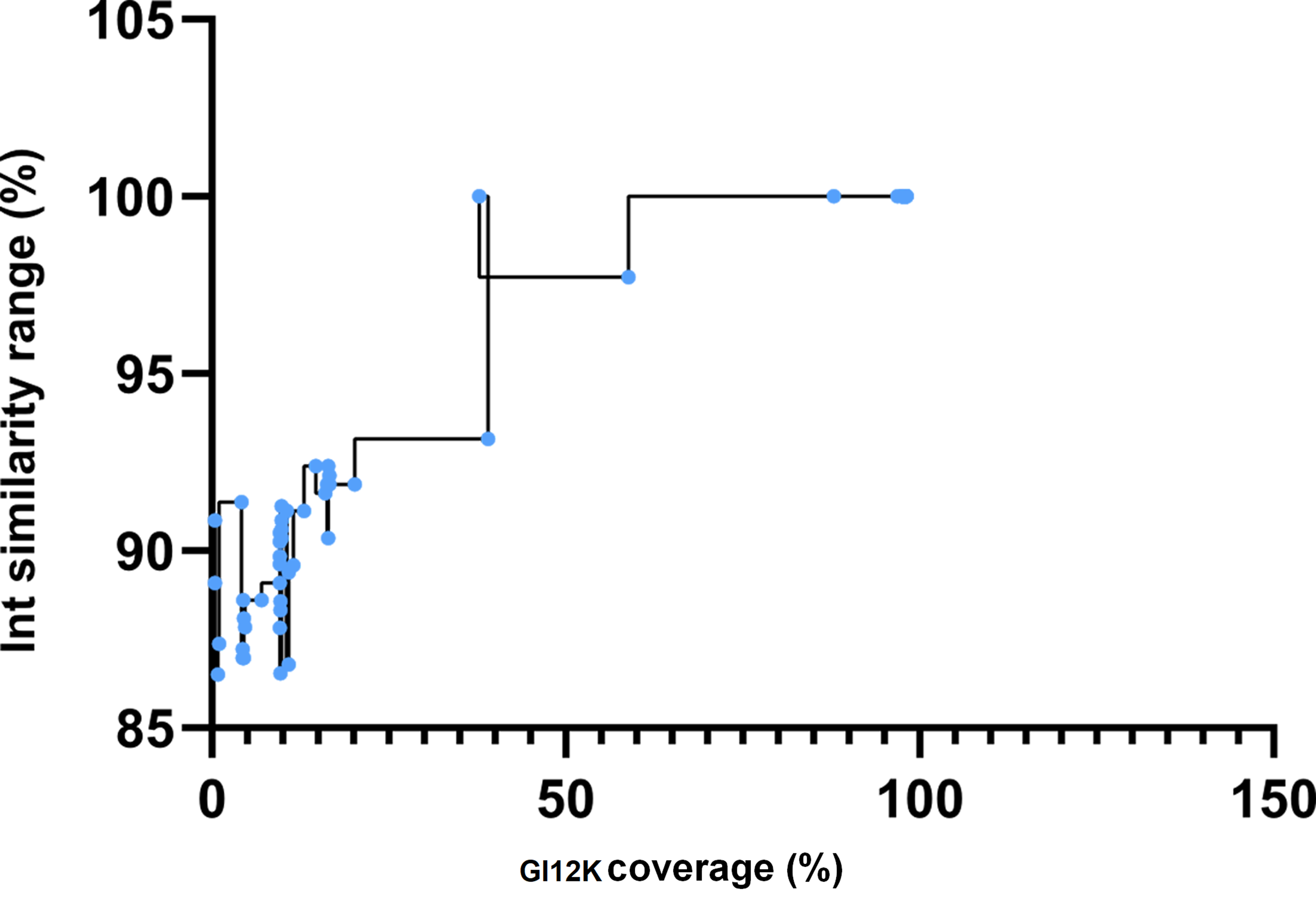

Supplement: Supplementary file 9 [file Image_5.TIF]
